# Neutrophil extracellular traps (NETs)-related lncRNAs signature for predicting prognosis and the immune microenvironment in breast cancer

**DOI:** 10.3389/fcell.2023.1117637

**Published:** 2023-02-02

**Authors:** Tongchao Jiang, Ying Wang, Xiaoyu Chen, Wen Xia, Shuyu Xue, Liwen Gu, Ling Guo, Huanxin Lin

**Affiliations:** ^1^ State Key Laboratory of Oncology in South China, Guangdong Key Laboratory of Nasopharyngeal Carcinoma Diagnosis and Therapy, Department of Radiotherapy, Collaborative Innovation Center for Cancer Medicine, Sun Yat-sen University Cancer Center, Guangzhou, Guangdong, China; ^2^ State Key Laboratory of Oncology in South China, Guangdong Key Laboratory of Nasopharyngeal Carcinoma Diagnosis and Therapy, Department of Medical Oncology, Collaborative Innovation Center for Cancer Medicine, Sun Yat-sen University Cancer Center, Guangzhou, Guangdong, China; ^3^ State Key Laboratory of Oncology in South China, Guangdong Key Laboratory of Nasopharyngeal Carcinoma Diagnosis and Therapy, Department of Nasopharyngeal Carcinoma, Collaborative Innovation Center for Cancer Medicine, Sun Yat-sen University Cancer Center, Guangzhou, Guangdong, China

**Keywords:** breast cancer, NETs, lncRNA, prognosis, tumor microenvironment, anticancer drugs

## Abstract

**Background:** Neutrophil extracellular traps (NETs) are closely associated to tumorigenesis and development. However, the relationship between NETs-related long non-coding RNAs (lncRNAs) and the characteristics of breast tumor remains an enigma. This study aimed to explore the clinical prognostic value of NETs-related lncRNAs, their correlation with the tumor microenvironment (TME) and their predictive ability of drug sensitivity in patients with breast cancer (BC).

**Methods:** The expression profiles of RNA-sequencing and relevant clinical data of BC patients were extracted from TCGA database. The co-expression network analysis, univariable, least absolute shrinkage and selection operator (LASSO) and multivariable Cox algorithms were employed to construct the NETs-related lncRNAs signature. A nomogram was established and validated to explore the clinical application. Furthermore, the immune microenvironment and drug sensitivity for BC with different prognostic risks were explored. Finally, the expression pattern of lncRNAs was validated using qRT-PCR in BC tissues and their adjacent non-cancerous tissues.

**Results:** Based on NETs-related lncRNAs, a prognostic risk model consisted of 10 lncRNAs (SFTA1P, ACTA2-AS1, AC004816.2, AC000067.1, LINC01235, LINC01010, AL133467.1, AC092919.1, AL591468.1, and MIR200CHG) was established. The Kaplan-Meier analysis showed that the overall survival (OS) was significantly better in low-risk BC patients than in high-risk BC patients (*P*_training cohort_ < 0.001, *P*_validation cohort_ = 0.009). The nomogram also showed good predictive accuracy for OS of BC individuals in both training and validation cohorts. The function enrichment analysis revealed that high-risk group was mainly enriched in immune-related functions and pathways, and the tumor mutation burden in this group was markedly higher than that in the low-risk group (*p* = 0.022). Moreover, significant differences were observed in immune cells, immune functions and immune checkpoint genes among BC patients at different risks (*p* < 0.05). The response to chemotherapeutic agents and immunotherapy were also closely related with the expression of NETs-related lncRNAs (*p* < 0.001). The expression of lncRNAs from experimental validation were generally consistent with the bioinformatics analysis results.

**Conclusion:** Our study provided a novel prognostic model for BC and yielded strong scientific rationale for individualized treatment strategies, elucidating immunotherapy in BC patients.

## Introduction

Breast cancer (BC) is the most prevalent and second most deadly malignancy in women, accounting for 31% of all newly diagnosed cancers (Siegel et al., 2022). At present, despite the advances in effective therapeutic strategies including surgical resection, endocrine therapy, and the combination of surgery with radiotherapy, chemotherapy and immunotherapy, BC still confronted with high morbidity, aggressiveness, metastasis, and recurrence rates (Pondé et al., 2019). Moreover, due to the remarkable tumor heterogeneity, breast cancers with the same subtypes can respond differently to therapy and have different prognosis (Pondé et al., 2019). Thus, specific molecular biomarkers and therapeutic targets for BC are pivotal elements to guide clinical practice.

Neutrophils, the most abundant endogenous immune effector cells, can respond to specific stimulation by releasing neutrophil extracellular traps (NETs), a type of regulated cell death termed “neutrophil extracellular traposis (NETosis)” (Ireland and Oliver, 2020). Primarily described as an antimicrobial mechanism for entrapping, constraining, and killing invading bacteria and other pathogens, NETs are complex extracellular networks composed of nuclear DNA fibers and mitochondria decorated with granular antimicrobial enzymes and histones (Papayannopoulos, 2018; Demkow, 2021). Subsequent studies have shown that NETs, forming a protective shield, have multiple pro-tumor capabilities, including primary growth and metastasis (Ireland and Oliver, 2020; Martins-Cardoso et al., 2020).

The long non-coding RNAs (lncRNAs), non-coding RNA longer than 200 nucleotides, do not directly participate in protein coding in cells, but are engaged in vital biological regulatory processes, including transcriptional regulation, mRNA processing regulation and mRNA post-transcriptional regulation (Yang et al., 2022). Recent finding indicated a role of lncRNA in regulating NETs in lung cancer (Wang et al., 2022). However, the lncRNAs associated with NETs in BC are less studied, and most lncRNAs regulating NETs have not been determined. Potential NETs-related biomarkers and prognostic biomarkers can be identified with the advances in high-throughput sequencing technologies and bioinformatics (Huang da et al., 2009).

Immune checkpoint blockade (ICB) therapy, relying on the immune tumor microenvironment, is an effective therapeutic strategy that blocks the immune checkpoint pathway to keep tumor cells from evading immune surveillance (Franzén et al., 2022). Unfortunately, most breast tumors with less tumor-infiltrating cytotoxic T cells and lower PD-L1 expression are usually considered as immune “cold” tumors and tend to have poorer efficacy with ICB therapy (Tekpli et al., 2019). Currently, a study has revealed that NETs have the ability to suppress T-cell responses in the tumor microenvironment through metabolism and functional exhaustion, thereby affecting immunotherapeutic efficacy (Kaltenmeier et al., 2021). Moreover, lncRNAs act as a key player in reshaping the immune landscape, regulating metabolic reprogramming, and functioning as a bond between tumor metabolism and anti-tumor immunity (Yang et al., 2022). Thus, exploring the interaction between NETs-related lncRNAs and tumor immune microenvironment can help to improve the understanding of the pathogenesis of “cold” breast cancer and offer potential therapeutic strategies for “cold” breast tumor.

In the current study, we first constructed a prognostic risk model composed of 10 NETs-related lncRNAs for BC patients based on public databases, and evaluated this risk model performance. Furthermore, the clinical significance and application value of the model and its effects on immune microenvironment and drug sensitivity were also explored. To the best of our knowledge, no previous studies have investigated the predictive value of NETs-related lncRNAs and their relationship with the immune microenvironment in BC. The present study identified NETs-related lncRNAs that may be potential therapeutic targets and prognostic and predictive markers for BC patients and could be used to further improve the treatment outcome of BC patients through individualized therapy.

## Materials and methods

### Data acquisition

The transcriptome RNA-seq data [fragments per kilobase of transcript per Million mapped reads (FPKM)] of 1,222 samples were obtained from the TCGA public database, including 1110 BC tissues and 112 normal adjacent tissues (https://portal.gdc.cancer.gov/repository). The corresponding clinicopathological data of BC were also downloaded from TCGA together. The protein-coding genes and lncRNAs were distinguished by applying the ensembl human genome browser GRCh38.p13. A total of 170 NETs-related genes were acquired from previously published studies (as shown in Supplementary Table S1) (Dwyer et al., 2014; Papayannopoulos, 2018). Then the correlation between the expression of NETs-related genes and corresponding lncRNAs was quantified by calculating the Pearson correlation coefficients. The strict criteria were used to identify NETs-related lncRNAs, with *p* < 0.001 and the absolute value of Pearson correlation coefficient more than 0.4 (|R| > 0.4).

### Determination of differentially expressed NETs-Related LncRNAs

The expression profiles of NETs-related lncRNAs of the 112 normal breast samples and 1110 BC samples were obtained, and the differential expression analysis was carried out with |Log_2_ fold change [FC]| >1 and false discovery rate (FDR) <0.05 using the “limma” and “pheatmap” R package. After deleting patients with incomplete information, 937 patients with breast cancer in total were randomly divided in an 8:2 ratio into training and validation cohorts for constructing and validating the NETs-related lncRNAs signature. Univariate Cox regression analysis for overall survival (OS) was employed to determine prognostic lncRNAs with *p* < 0.05 in the training cohort. The least absolute shrinkage and selection operator (LASSO) Cox regression algorithm was performed to lessen the chance of overfitting. Furthermore, multivariate Cox regression analysis was applied to calculate the regression coefficient of the prognostic risk score model.

### Construction of the LncRNA-mRNA co-expression network

The lncRNA-mRNA co-expression network was constructed to demonstrate the correlation between the NETs-related lncRNAs and their corresponding mRNAs, and visualized using the Cytoscape software (version 3.7.2, http://www.cytoscape.org/).

### Construction and validation of NETs-related LncRNAs signature

The risk score for each BC patient in both the training and validation cohorts was calculated according to the following equation:

$$Risk\;score=\overset{\infty }{\underset{i=1}{\sum }}coefi\times xi$$ (1)

where coefi is the regression coefficient and xi is the corresponding lncRNA expression level. The BC patients in the training cohort were separated into high- and low-risk groups based on the median risk score. The same median score was used to divide patients in the validation cohort into high- and low-risk groups. The Kaplan-Meier method, risk score heatmap, distribution of risk score and survival status were used to assess the validity of the prognostic risk model by applying the “survivalROC” and “pheatmap” R package.

Univariate and multivariate Cox regression algorithm were conducted to assess the prognostic significance of risk scores based on other clinical parameters (age, stage, and subtype) in the training cohort. The receiver operating characteristic (ROC) curves and C-index were performed to evaluate the predictive power of this signature. Then, a nomogram was constructed based on these parameters as implemented in the “rms” R package. The validity of prognostic model in their accuracy of prediction for 1-, 3-, and 5-years OS was further assessed by the calibration curves and time-dependent receiver operating characteristic (ROC) curves in the training and validation cohorts.

### Functional enrichment analysis

To investigate the potential biological functions of the 10 NETs-related lncRNAs in BC, the gene set enrichment analysis (GSEA) was conducted to look for the tumor hallmarks associated with risk scores. The FDR <0.25 and *P* adjusted value <0.05 were adopted as the criteria for statistical significance. An enrichment lot was performed to visualize the top five functions enriched by two groups. After that, the differentially expressed genes between the two risk scores groups were determined by differential expression analysis of two groups with |log FC| >1 and *p* < 0.05. The pathway enrichment analysis for Gene ontology (GO) and kyoto encyclopedia of genes and genomes (KEGG) on the screened genes were conducted by employing the “clusterProfiler” R package. Then, the “enrichplot” and “ggplot2” R packages were applied to visualize the enrichment results.

### Comprehensive analysis of molecular variation and immune infiltration

The 977 single nucleotide variants (SNVs) data were downloaded in the TCGA public database and the “Maftools” R package was applied to calculate the tumor mutation burden (TMB) for each BC patient. The correlation between the risk score and TMB was analyzed using the Spearman’s algorithm. The Kaplan-Meier survival analysis was performed for different TMB groups. Furthermore, the CIBERSORT deconvolution algorithm with 1,000 permutations was applied to calculate the abundance of immune cell infiltration in the tumor immune microenvironment of high- and low-risk patients for BC with *p* < 0.05.

### Evaluation of the immune cell infiltration, immune function, and immune checkpoint genes

Single-sample Gene Set Enrichment Analysis (ssGSEA) was performed to determine the biological function differences between the high- and low-risk groups by applying the “GSVA” R package. The potential immune checkpoint genes were chosen according to previous published literature. The different expression levels of immune checkpoint genes between risk groups were analyzed by the Wilcoxon test.

### Evaluation of drug sensitivity and immunotherapy efficacy

To assess the therapeutic efficacy of chemotherapy and targeted agents in constructing prognostic risk signature of NETs-related lncRNAs, the half inhibitory concentration (IC_50_) of chemotherapeutic and target therapeutic drugs were calculated using the “ggplot2” and “pRRophetic” R packages. The Wilcoxon signed-rank method was applied to compare the IC_50_ of different risk groups. Then, immunotherapy responsiveness was predicted in different risk groups by applying the Tumor Immune Dysfunction and Exclusion (TIDE) online Tool (http://tide.dfci.harvard.edu/).

### Tissue specimens and quantitative real-time polymerase chain reaction

A total of 10 matched pairs of tumor specimens and adjacent normal tissues were obtained from BC patients who underwent tumor resection. All tissue specimens were collected from the Breast Surgery Department of Sun Yat-sen University Cancer Center, Guangzhou, China, with patients’ consent and approval from the Medical Ethics Committee of the hospital.

For RNA expression assay, total RNA was extracted using TRIzol reagent (Invitrogen, 15596018) and quantified using a Nanodrop (Thermo Scientific). Then, cDNA was obtained using the PrimeScript™ RT Reagent Kit (Takara, RR036A) according to the manufacturer’s instruction. Quantitative real-time polymerase chain reaction (qRT-PCR) was performed with the TB Green™ Premix Ex Taq™ (TaKaRa, RR420A) using SYBR Green (Roche) on a LightCycler 480 (Roche). The relative abundance of each lnRNA, using GAPDH as an endogenous control, was calculated by 2^−∆∆CT method. The primers sequences used in this study are listed in Supplementary Table S2. Analysis between the two groups was performed by paired-sample t-tests. *p* < 0.05 was considered statistically significant.

## Results

### Identification of differentially expressed LncRNAs related to NETs

**FIGURE 1:**
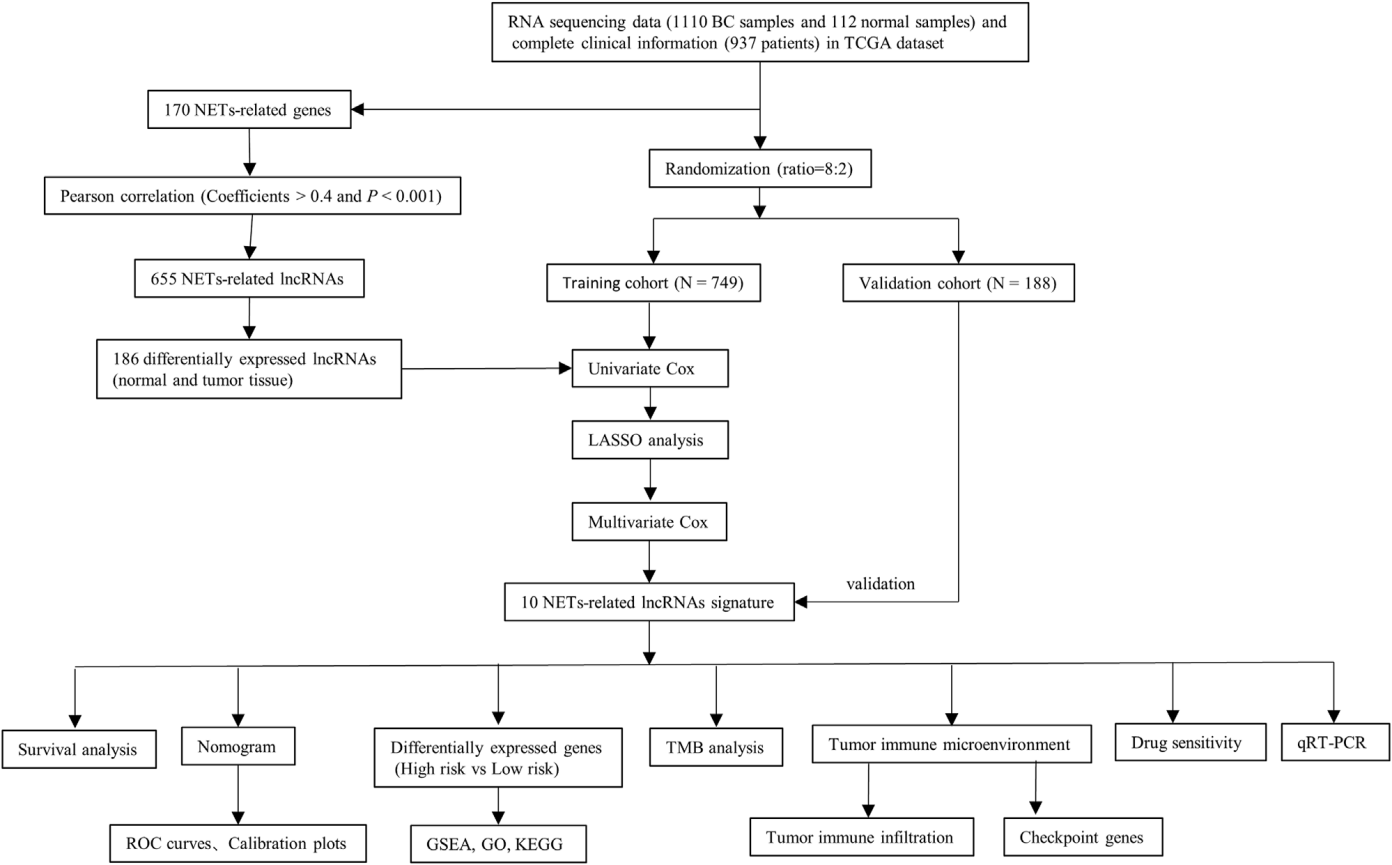
Flow diagram of the study.

**FIGURE 2:**
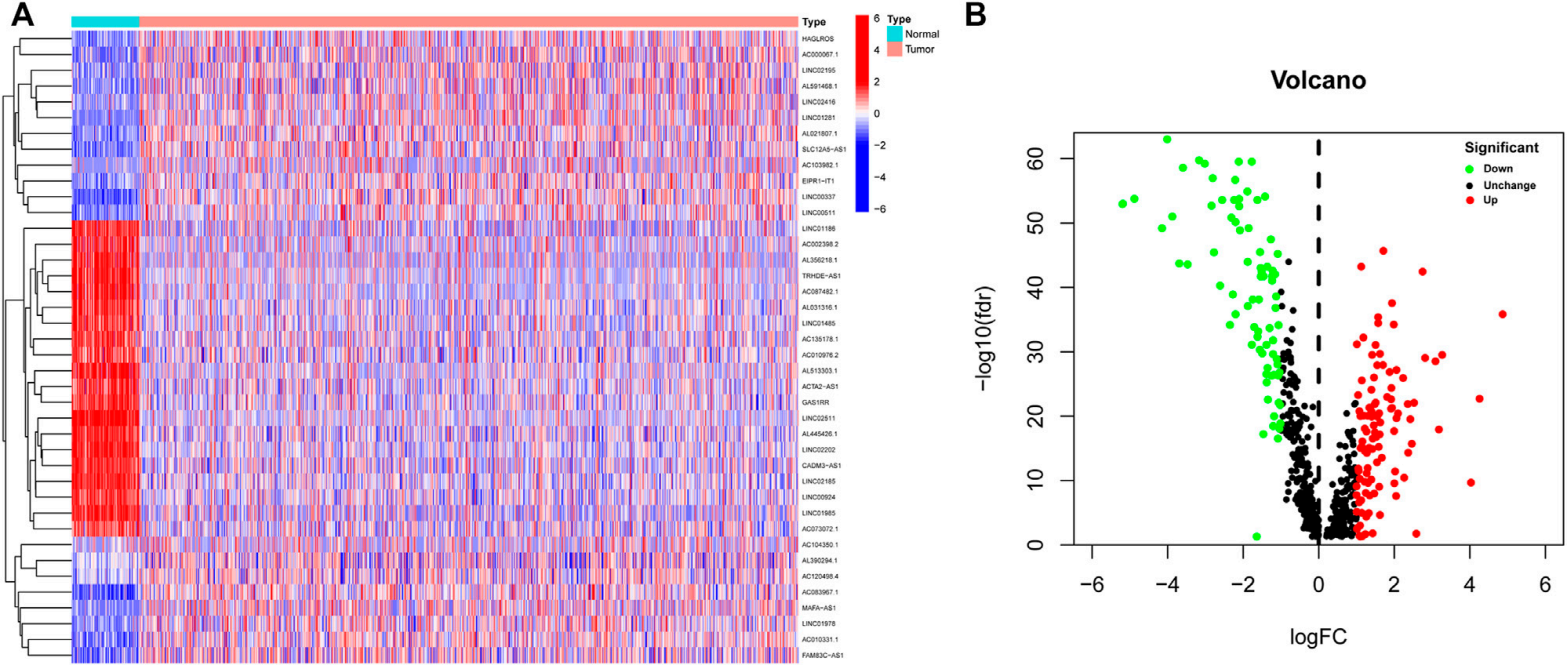
Identification of differentially expressed NETs-related lncRNAs in BC. **(A)** Heatmap demonstrating the differential expression of the top 20 up-regulated and 20 down-regulated NETs-related lncRNAs in BC tissues and normal tissues. **(B)** Volcano plot exhibiting 105 up-regulated and 81 down-regulated NETs-related lncRNAs. NETs, neutrophil extracellular traps; lncRNAs, long non-coding RNAs; BC, breast cancer.

A flow chart of the study is depicted in Figure 1. A total of 14,142 lncRNA transcripts and 19,658 protein-coding genes were identified from the TCGA database. The gene expression of NETs in BC was screened by matching the mRNA expression matrix of TCGA and 170 NETs-related genes. Afterwards, 655 lncRNAs were identified to be highly correlated with the expression of NETs-related genes (|R| > 0.4 and *p* < 0.001), among which 186 lncRNAs (as shown in Supplementary Table S3) were identified based on differential expression between normal and tumor tissue. A heatmap was performed to demonstrate the differential expression of the top 20 up-regulated and 20 down-regulated lncRNAs (Figure 2A), and volcano plot of differentially expressed lncRNAs showed 105 up-regulated lncRNAs and 81 down-regulated lncRNAs in BC (Figure 2B).

### Identification of prognostic NETs-Related LncRNAs and establishment of risk model

**FIGURE 3:**
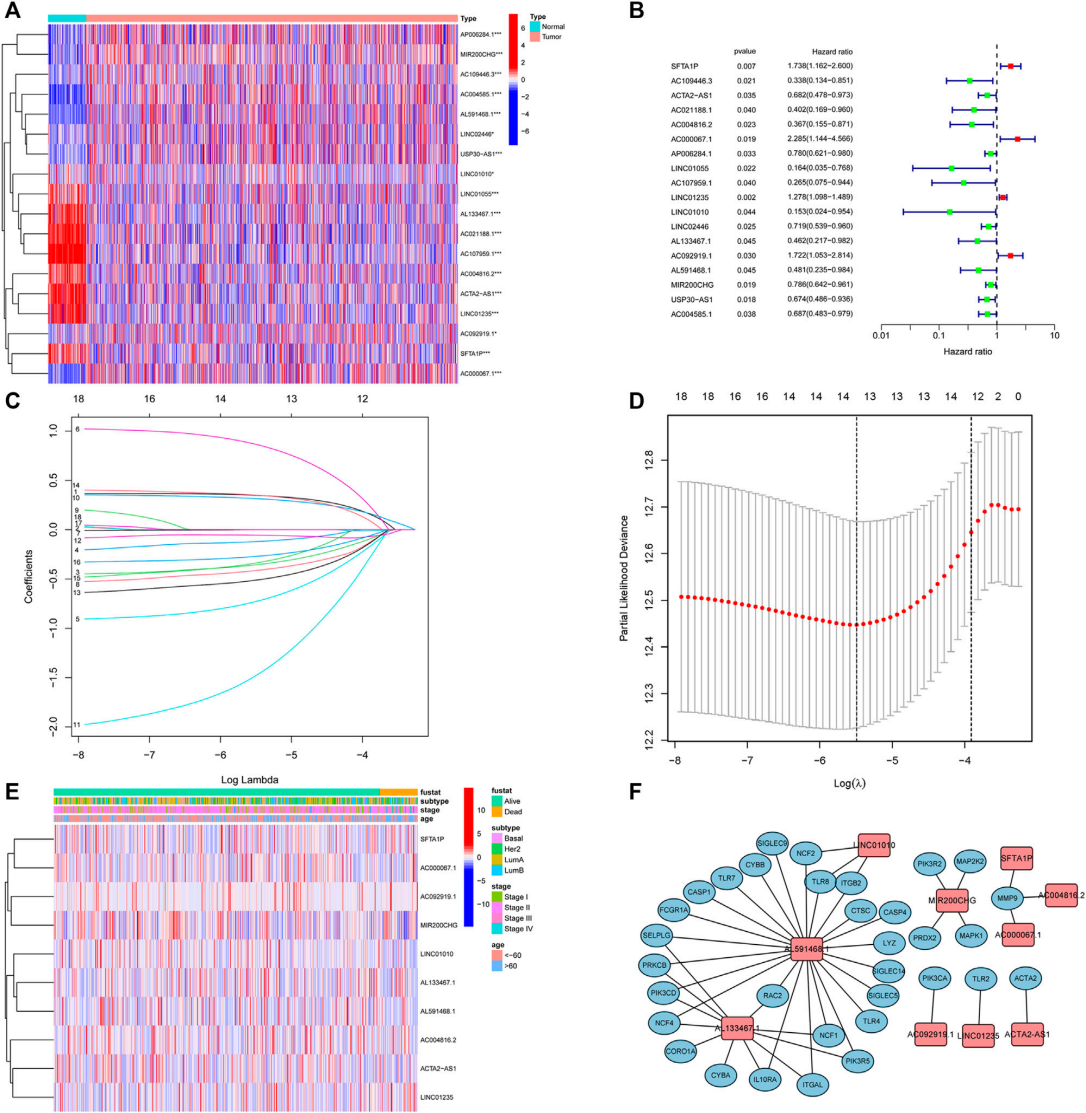
Identification of prognostic NETs-related lncRNAs. **(A)** Heatmap of OS-associated differentially expressed NETs-related lncRNAs. **(B)** Forest plot of NETs-related lncRNAs associated with BC prognosis *via* univariate analysis. **(C)** LASSO coefficient profiles of 13 NETs-related lncRNAs. **(D)** Cross-validation for tuning parameter selection in the proportional hazards model. **(E)** Heatmap of the 10 NETs-related lncRNAs *via* multivariate analysis. **(F)** Correlation between 10 NETs-related lncRNAs and NETs genes. NETs, neutrophil extracellular traps; lncRNAs, long non-coding RNAs; BC, breast cancer; OS, overall survival.

Next, to establish a convincing risk predictive model, BC patients were randomly divided in an 8:2 ratio into training (*n* = 749) and validation cohorts (*n* = 188). In the training cohort, a univariate Cox regression algorithm was performed to determine the lncRNAs related to BC patient survival and 18 prognosis-related candidate lncRNAs were filtered out (Figures 3A, B). The 18 lncRNAs were incorporated into the LASSO Cox regression algorithm to improve model accuracy and reduce model overfitting (Figure 3C), and cross-validation was performed and 13 prognostic lncRNAs were screened out (Figure 3D). To further improve clinical utility, 13 lncRNAs signatures were purified by performing stepwise multivariate Cox regression analysis (Figure 3E). Then, a prognostic risk model was constructed based on the correlation coefficient of the expression of lncRNAs in the multivariable Cox regression model. The following formula was applied to determine the risk score: risk score = (0.366 × SFTA1P expression level) + (−0.500 × ACTA2-AS1 expression level) + (−0.907 × AC004816.2 expression level) + (1.013 × AC000067.1 expression level) + (0.361 × LINC01235 expression level) + (−2.069 × LINC01010 expression level) + (−0.685 × AL133467.1 expression level) + (0.387 × AC092919.1 expression level) + (−0.676 × AL591468.1 expression level) + (−0.377 × MIR200CHG expression level). The correlation between the 10 prognostic lncRNAs and NETs-related genes was shown in Figure 3F.

### Validation of the prognostic risk model

**FIGURE 4:**
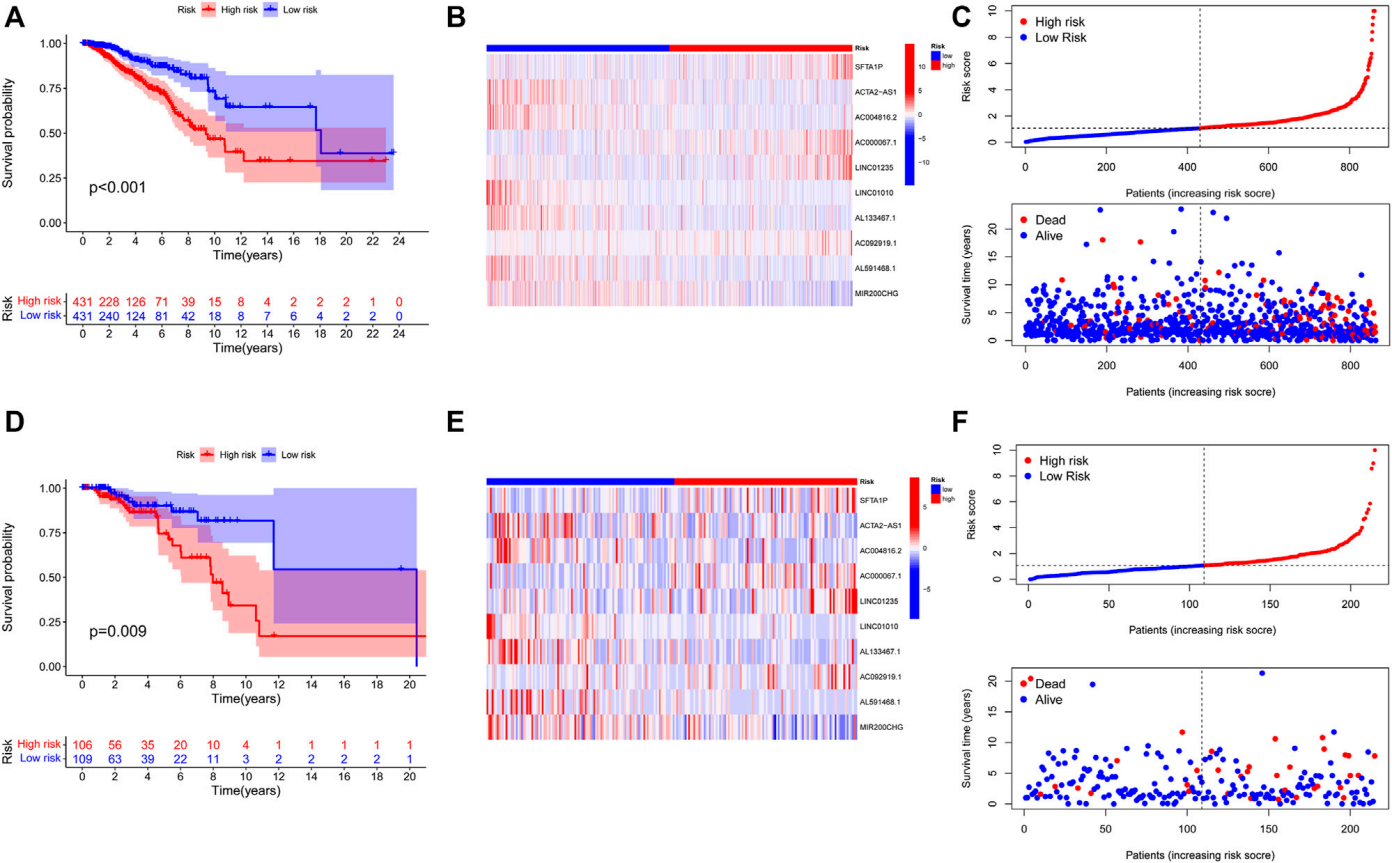
Construction and validation of prognostic signatures of NETs-related lncRNAs. **(A-D)** Kaplan–Meier curves of overall survival for BC patients based on the risk score in the training **(A)** and validation **(D)** cohorts. **(B-E)** Heatmap of the signature of 10 NETs-related lncRNAs in the training **(B)** and the validation **(E)** cohorts. **(C-F)** Distribution of risk score, OS, and OS status in the training **(C)** and the validation **(F)** cohorts. NETs, neutrophil extracellular traps; lncRNAs, long non-coding RNAs; BC, breast cancer; OS, overall survival.

Initially, The BC patients in the training and validation cohorts were categorized into high- and low-risk groups according to the training cohort’s medium risk score. The validation cohort was used for internal validation to weight the predictive capability of the model. As represented by the Kaplan–Meier curves, a significantly inferior OS was observed for those in the high-risk group in comparison with those in the low-risk group in both the training (*p* < 0.001) (Figure 4A) and validation cohorts (*p* = 0.009) (Figure 4D). The visualized heatmap revealed differential expression of lncRNAs in high- and low-risk groups in training and validation cohorts (Figures 4B, E). Along with the increase in the risk score, the proportion of patients in the high-risk group also increased, and so did the level of mortality (Figures 4C, F).

### Construction and assessment of a clinical prognostic model

**FIGURE 5:**
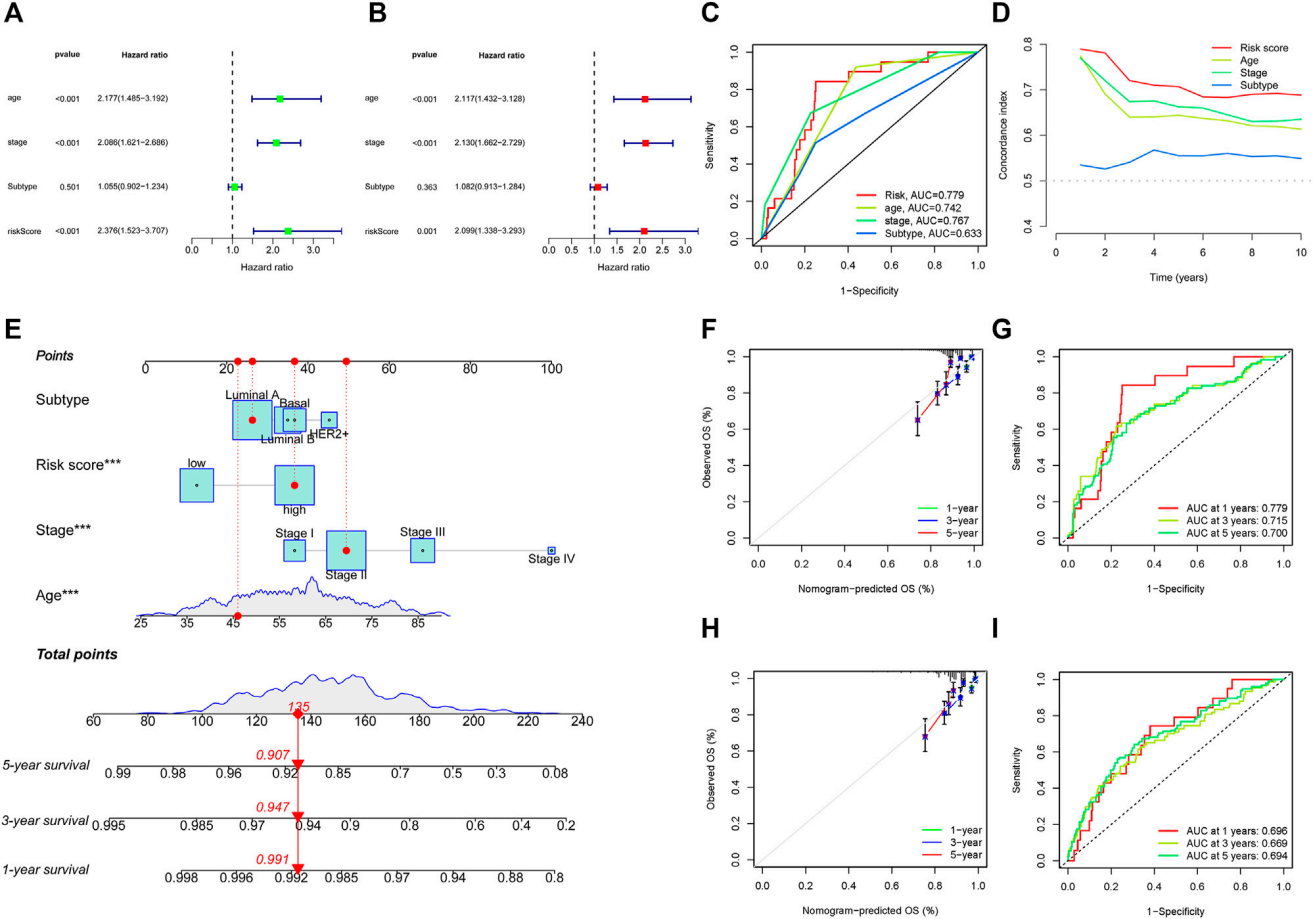
Identification of prognostic parameters in BC. **(A)** The univariate Cox regression analysis in training cohort. **(B)** The multivariate Cox regression analysis in training cohort. **(C, D)** ROC curves and C-index for the risk score, age, stage, and subtype in training cohort. **(E)** Construction of nomogram for survival prediction in training cohort. **(F, I)** Calibration plots and ROC curves of the nomogram for predicting the probability of OS at 1-, 3-, and 5- years in training **(F, G)** and validation **(H, I)** cohorts. ROC, receiver operating characteristic; BC, breast cancer.

To further evaluate the possibility that the risk score could serve as an independent BC prognostic signature, the univariate Cox regression analysis was performed by matching the risk score of BC patients in the training cohort with conventional clinicopathological parameters (age, stage, and subtype). In the group of 749 individuals included in this investigation, age, stage, and risk score were associated with the prognosis of the BC patients (Figure 5A). Considering the impact of molecular subtype on BC survival, a multivariable Cox algorithm was conducted to further screen out four prognostic factors of the BC patients (age, stage, subtype, and risk score) (Figure 5B), which was consistent with the result of the univariate Cox regression analysis. The ROC curves and C-index also revealed that the risk score acted as an important role in predicting BC prognosis in training cohort (Figures 5C, D). Based on the four parameters, a nomogram was conducted to predict an individual’s prognosis at 1-, 3-, and 5- years (Figure 5E). The calibration curves showed high consistency between the predicted and the actual 1-, 3- and 5-years survival probabilities (Figure 5F). ROC curves analysis also showed satisfactory AUC values at 1-, 3- and 5- years (0.779, 0.715, and 0.700, respectively) (Figure 5G). In addition, the predictive power and performance of this nomogram was also confirmed in the validation cohort, with better calibration curves and AUC values (Figures 5H, I).

### Molecular characteristics of different risk groups

**FIGURE 6:**
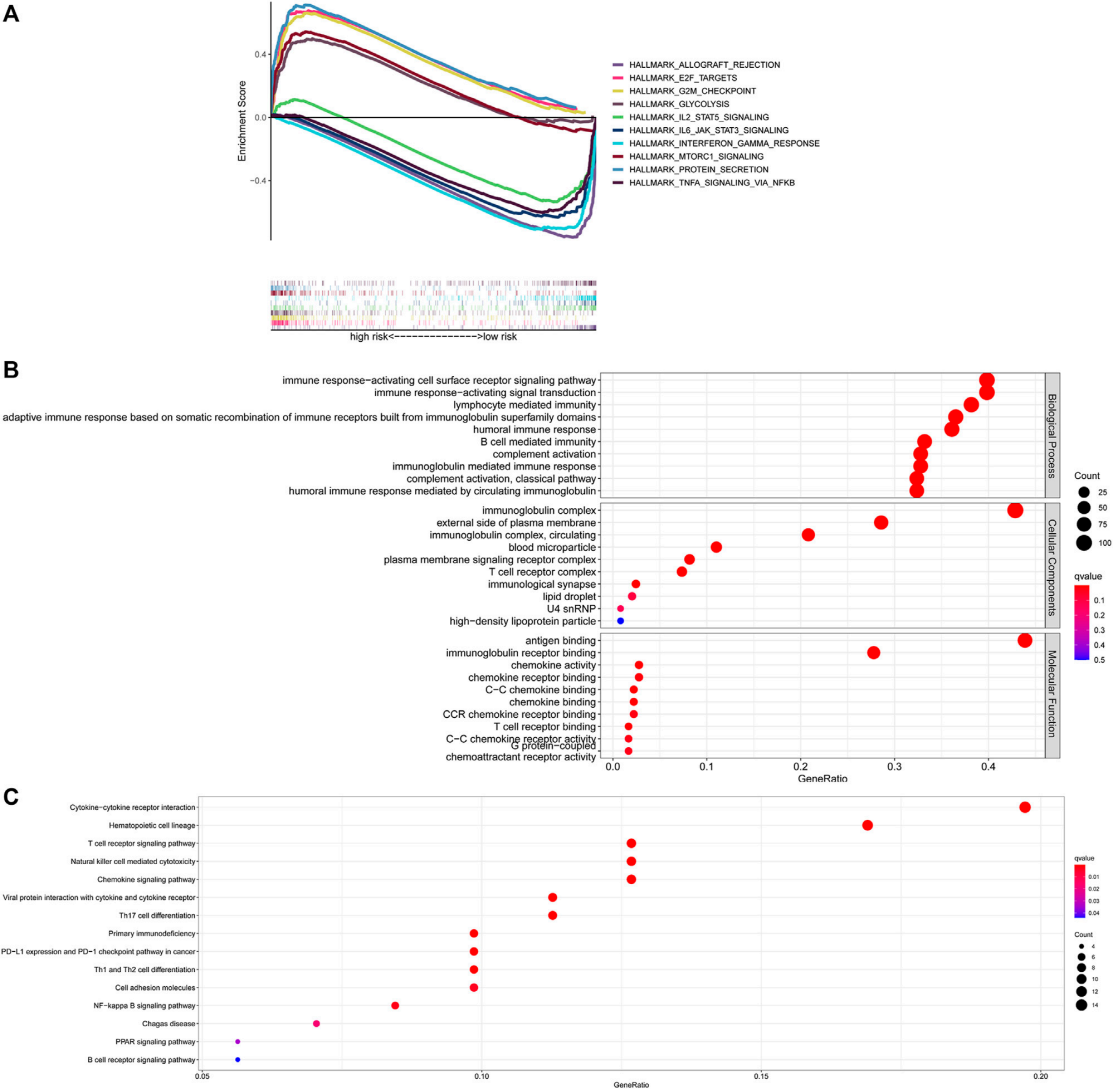
Exploration of the possible functions and pathways of NETs-related lncRNAs. **(A)** Tumor hallmarks in high- and low-risk subgroups. **(B)** GO analysis of different expression gene sets between different risk subgroups. **(C)** KEGG analysis of different expression gene sets between different risk subgroups. NETs, neutrophil extracellular traps; lncRNAs, long non-coding RNAs.

To gain further insight into the specific molecular differences between high- and low-risk groups, differentially expressed genes (as shown in Supplementary Table S4) were identified and functional annotation was conducted by the GSEA. The GSEA with | ES scores | > 0.5, FDR <0.25 and *P* adjusted value <0.05 for the five most significant pathways showed enrichment of tumor hallmarks in high- and low-risk subgroups. The high-risk group was enriched in HALLMARK_PROTEIN_SECRETION, HALLMARK_GLYCOLYSIS, HALLMARK_G2M_CHECKPOINT, HALLMARK_MTORC1_SIGNALING, HALLMARK_E2F_TARGETS, while the low-risk group was enriched in HALLMARK_ALLOGRAFT_REJECTION, HALLMARK_IL2_STAT5_SIGNALING, HALLMARK_TNFA_SIGNALING_VIA_NFKB, HALLMARK_INTERFERON_GAMMA_RESPONSE, HALLMARK_IL6_JAK_STAT3_SIGNALING (Figure 6A). Then, the potential functions and pathways were identified using GO and KEGG enrichment analyses. GO analysis showed that the differentially expressed genes between the risk score subgroups were enriched in immune response−activating cell surface receptor signaling pathway and immune response−activating signal transduction (Biological Process), immunoglobulin complex and external side of plasma membrane (Cellular Components), antigen binding and immunoglobulin receptor binding (Molecular Function; Figure 6B). KEGG analysis showed that the differentially expressed genes were enriched in cytokine−cytokine receptor interaction, hematopoietic cell lineage, T cell receptor signaling pathway, natural killer cell mediated cytotoxicity, chemokine signaling pathway (Figure 6C). These results indicated that enrichment was mainly focused on immune-related functions and pathways.

### Relationship between risk score and TMB

**FIGURE 7:**
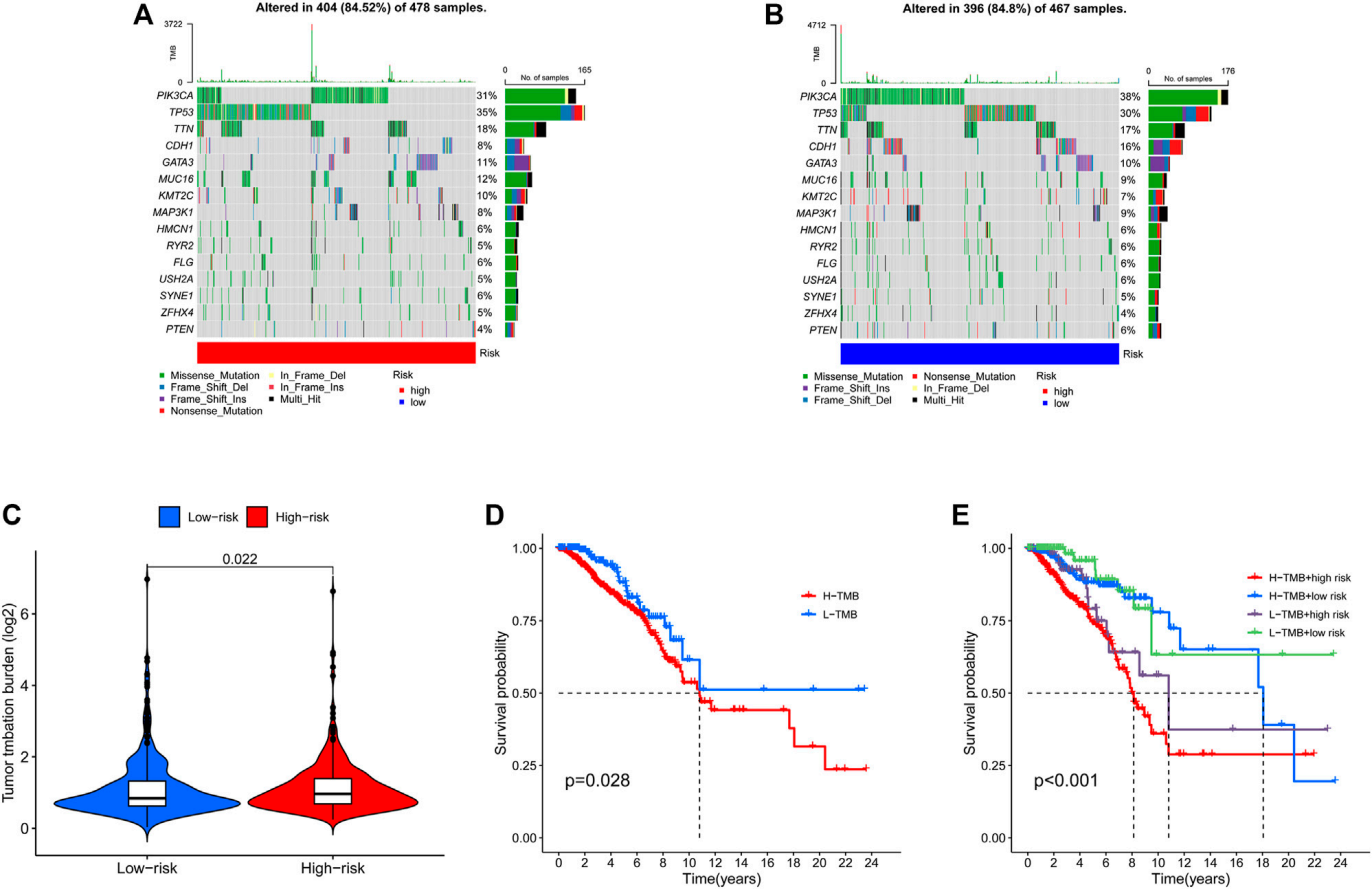
Landscape of mutation profiles in high- and low-risk BC patients. **(A)** The TMB in high-risk BC patients. **(B)** The TMB in low-risk BC patients. **(C)** The difference of TMB was significant in BC patients with different risk. **(D)** The lower TMB was positively correlated with the OS in BC patients. **(E)** Comparison of OS in BC patients with different risks and different TMBs. BC, breast cancer; TMB, Tumor mutation burden; OS, overall survival.

**FIGURE 8:**
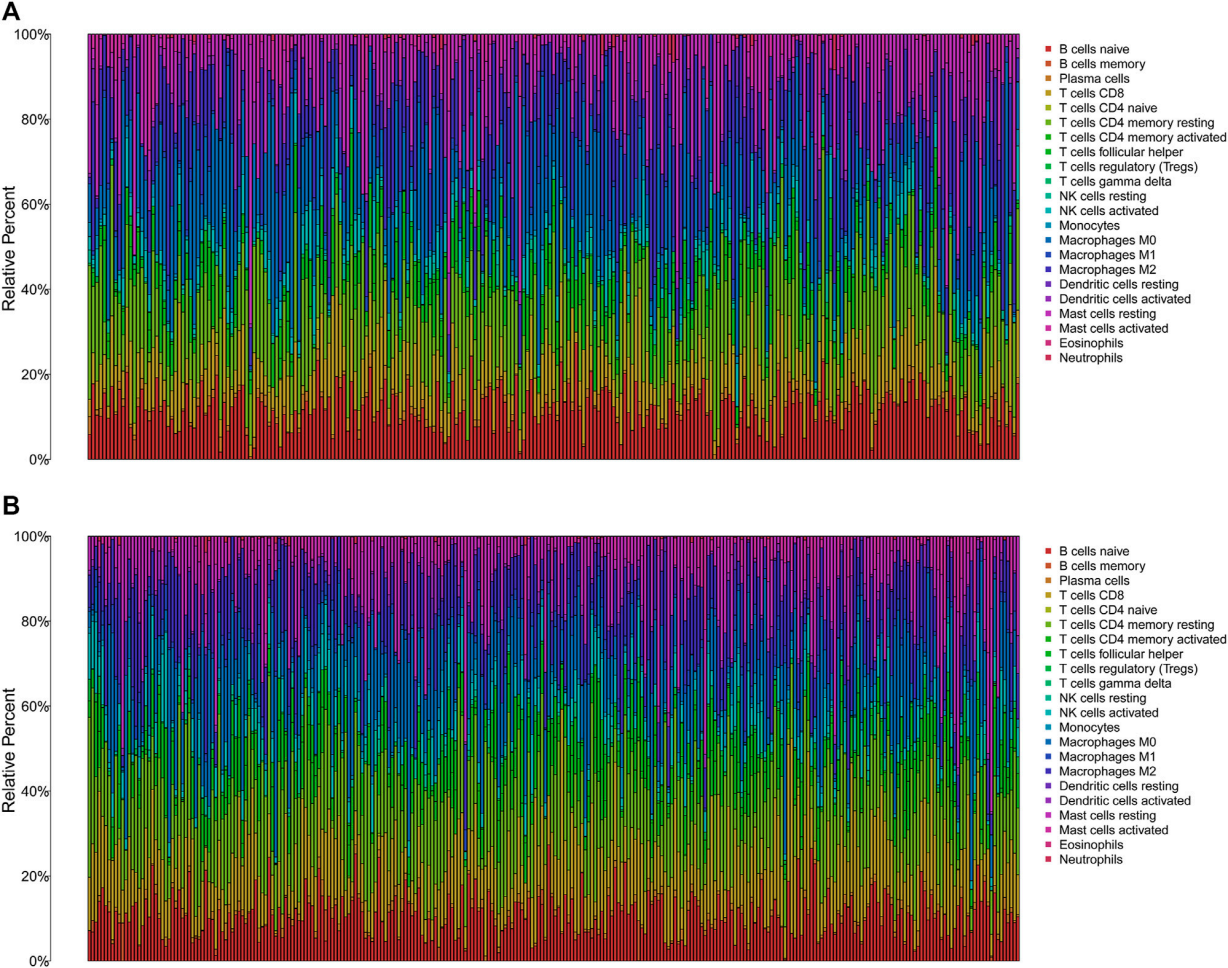
The immune infiltration of 22 immune cell types in high- and low-risk patients with BC. **(A)** The immune infiltration of 22 immune cell types in high-risk patients with BC. **(B)** The immune infiltration of 22 immune cell types in low-risk patients with BC. BC, breast cancer.

The TMB has been implicated as a biomarker for responses to immune checkpoint inhibitor therapy (Chan et al., 2019). The TMB of the high- and low-risk groups of BC patients was shown in Figures 7A, B. The difference in TMB between the two groups was statistically significant (*p* = 0.022, Figure 7C). Moreover, the patients in the low-TMB group experienced significantly better survival than those in the high-TMB group (*p* = 0.028, Figure 7D). Moreover, special subgroup analyses stratifying samples were performed according to the combination of TMB status and risk groups. The result showed that some patients with high-risk score in the low- or high-TMB groups had significantly shorter OS than those with low-risk score in the low- or high-TMB groups (*p* < 0.001, Figure 7E), but no significance could be calculated in the different TMB groups with the same risk scores. Thus, a high level of TMB, commonly considered as a “universal marker,” might fail to accurately predict the reactivity of checkpoint inhibitors across all cancer types. The exploration of the tumor microenvironment (TME) based on the risk predictive model was further conducted, and the proportion of 22 types of tumor-infiltrating immune cells in the high-risk and low-risk groups are shown in Figures 8A, B.

### Immune status and immune function in the different risk groups

**FIGURE 9:**
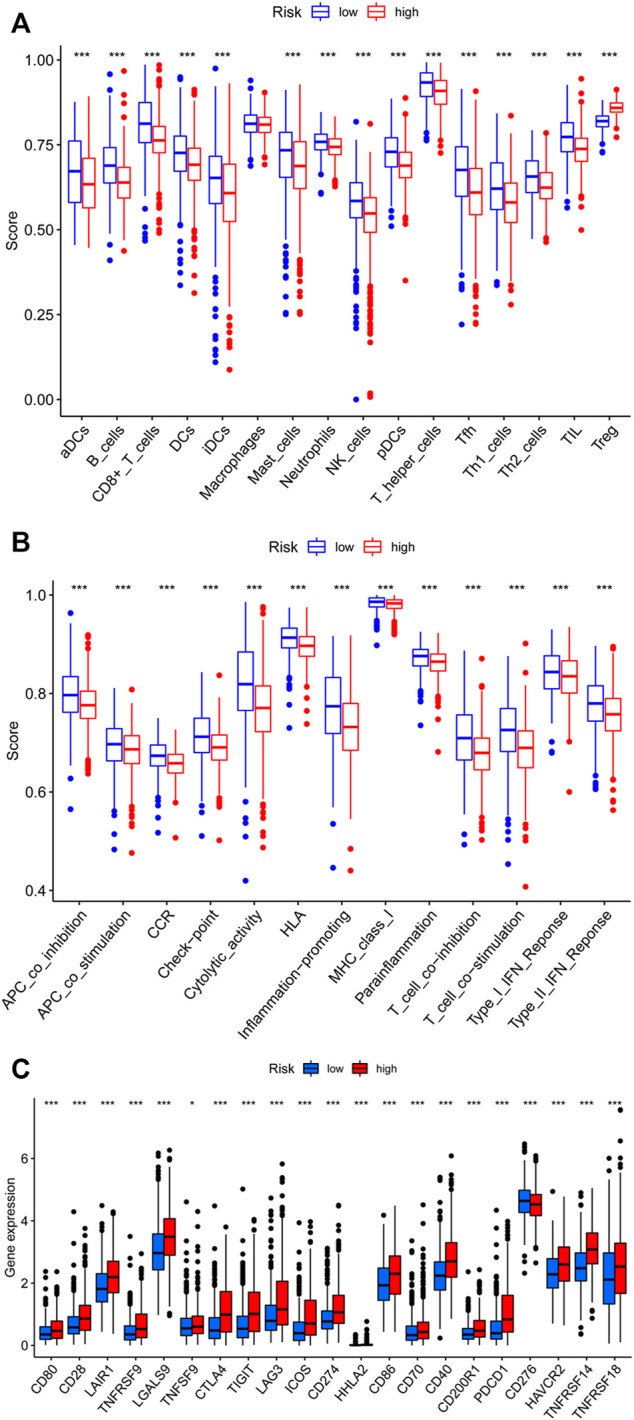
Assessment of Immune Cell Infiltration, Immune Function Signature, and Immune Checkpoint Genes in Different risk Groups. **(A)** Differences in the infiltration of immune cells between the high- and low-risk groups. **(B)** Differential expression levels of immune function signature between the high- and low-risk groups. **(C)** Differential expression of immune checkpoint genes between the high- and low-risk groups. ns, not significant, *p < 0.05, **p < 0.01, ***p < 0.001.

Then, we analyzed the potential correlation between NETs-related lncRNAs and 16 immune cells and the scores of 13 immunological functions using the ssGSEA algorithm. The results revealed that activated dendritic cells (aDCs), B cells, CD8 T cells, dendritic cells (DCs), immature Dendritic Cells (iDCs), mast cells, neutrophils, natural killer cells, plasmacytoid dendritic cells (pDCs), T follicular helper cell, type1 T helper cells, type2 T helper cells, tumor-infiltrating lymphocyte were more predominant in the low-risk group, while regulatory cells (Tregs) were more abundant in the high-risk group (Figure 9A). In addition, immune-related functions were highly enriched in the low-risk group (*p* < 0.01, Figure 9B). It has also been reported that blocking the immune checkpoint pathway is an extremely promising approach to achieve anti-cancer immunity (Li et al., 2019). Therefore, we made a comparison in the expression discrepancies of checkpoint genes between the high- and low-risk groups. The result was shown in Figure 9C, which indicated that there was significant difference in the expression of all checkpoint genes between the two groups, with all immune checkpoint molecules except CD276 being highly expressed in the high-risk group. These results demonstrate that NETs-related lncRNAs signature can be applied to evaluate the tumor immune microenvironment and the expression of immune checkpoint genes in BC patients.

### Prediction of chemotherapy efficacy and immunotherapy response

**FIGURE 10:**
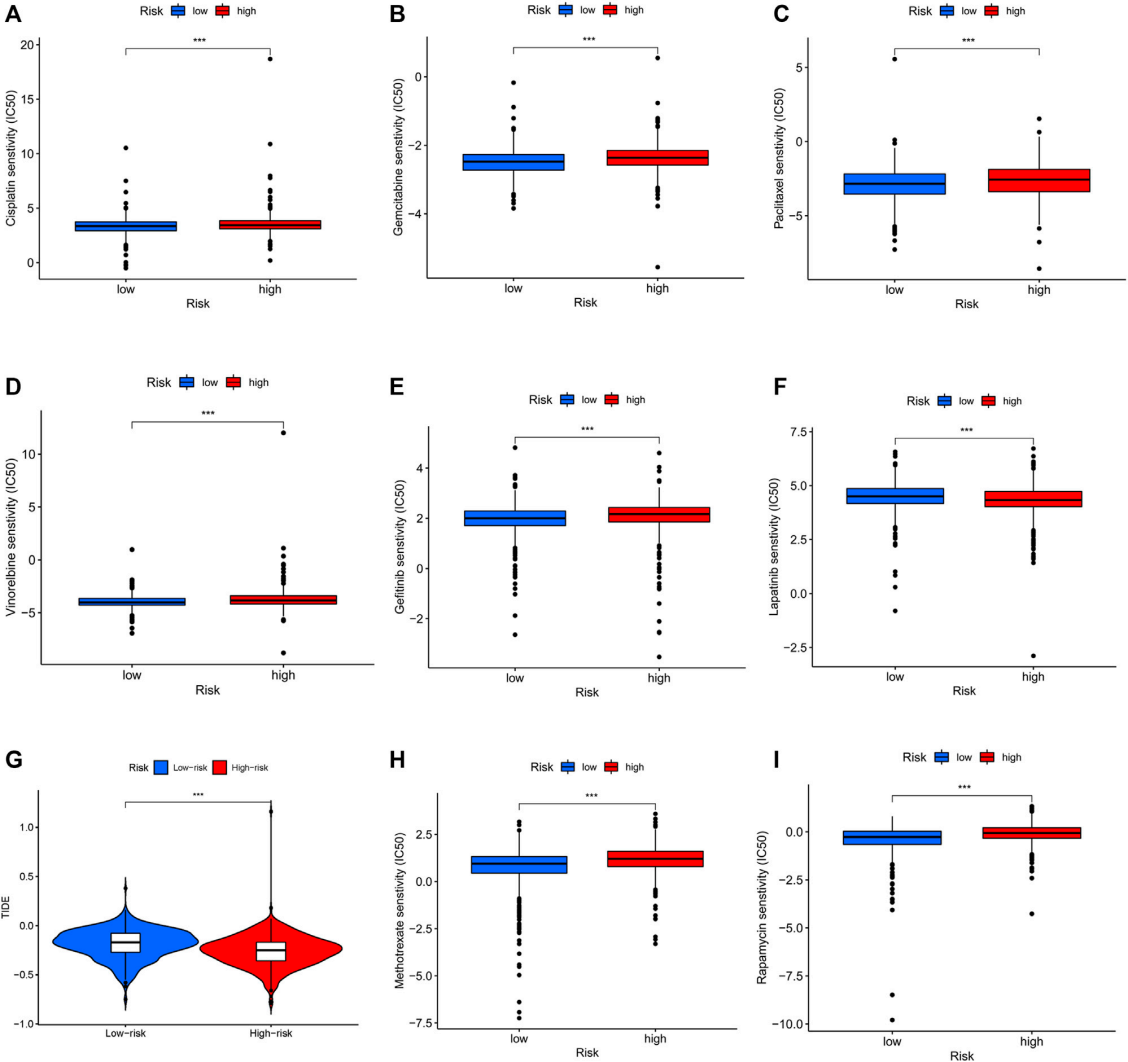
Differential sensitivity to chemotherapies and immunotherapy between the high- and low-risk patients with BC. BC, breast cancer. ***p < 0.001.

To assess the prediction performance of NETs-related lncRNAs on drug therapy for BC, we performed an analysis of the relationship between high- and low-risk groups and the efficacy of commonly used therapeutic agents using the “pRRophetic” R package. Our study suggested that the high-risk group showed significant correlation with a higher IC_50_ for chemotherapeutic and targeted agents such as cisplatin, gemcitabine, paclitaxel, vinorelbine, and gefitinib, which might indicate low-risk group was more suitable for these agents (all *p* < 0.001, Figures 10A–E). Conversely, lapatinib is more suitable for the high-risk group with a lower IC_50_ (*p* < 0.001, Figure 10F). In immunotherapy, TIDE scores were applied to assess the efficacy of immune checkpoint (PD-1 and CTLA-4) inhibitors in the high- and low-risk groups. High-risk patients had significantly lower TIDE scores compared to low-risk patients (*p* < 0.001, Figure 10G), indicating that patients in the high-risk group might have better response when receiving immune therapy. In the era of immunotherapy, the focus on treatment efficacy should be accompanied by a focus on the management of immune-related adverse effects. The commonly used immunosuppressive agents, such as methotrexate and rapamycin, differed significantly between the two groups, with the high-risk group having a higher IC_50_ (*p* < 0.001, Figures 10H, I).

### Exploration and verification of signature-related LncRNAs expression profiles

**FIGURE 11:**
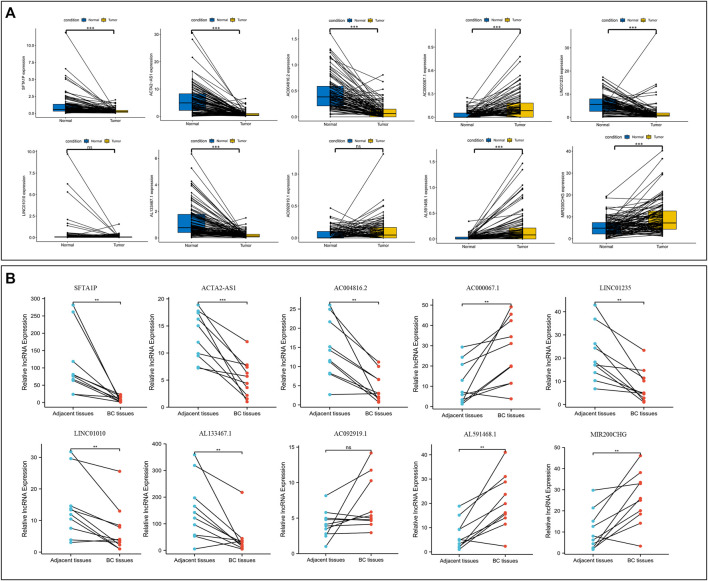
Validation the expression level of NETs-related lncRNAs. **(A)** The differential expression of 10 NETs-related lncRNAs between tumors and their matched benign tissue in TCGA RNA-seq data. **(B)** The expression levels of 10 NETs-related lncRNAs in 10 paired samples of BC tumor and adjacent normal tissues were examined by qRT-PCR. ns, not significant, *p < 0.05, **p < 0.01, ***p < 0.001. NETs, neutrophil extracellular traps; lncRNAs, long non-coding RNAs; BC, breast cancer.

First, the paired lncRNAs expression profiles of breast samples were analyzed by TCGA databases. Compared with adjacent normal breast tissues, the lncRNAs expression of SFTA1P, ACTA2-AS1, AC004816.2, LINC01235, and AL133467.1 were lower, while the expression of AC000067.1, AL591468.1, and MIR200CHG was higher in BC tissues (Figure 11A). Only LINC01010 and AC092919.1 were not statistically significant in paired tissues. To further confirm the validity of this model, the expression levels of all lncRNAs in 10 BC tumor tissues and matched normal tissues from our hospital were detected using qRT-PCR. Except for LINC01010, their expression trend was consistent with the bioinformatic analysis results (Figure 11B). The qRT-PCR results indicated that our bioinformatics analysis was accurate and reliable, reinforcing our conclusions from these data.

## Discussion

Recent Studies have shown that the prognosis and therapeutic response in tumor patients can vary according to molecular characteristics, although patients share homogeneous clinicopathological risk variables (Chan et al., 2019). Thus, novel molecular prognostic markers need to be identified to complement the clinical parameters to predict prognosis. NETs play a critical role in the tumor microenvironment and contribute to tumor migration, invasion, and distant metastasis in different ways (Ireland and Oliver, 2020; Martins-Cardoso et al., 2020; Demkow, 2021). Functionally, NETs function as a physical barrier to shield tumor cells from interactions with neighboring anti-tumor immune cells such as NK cells and CD8^+^ T cells in the tumor immune microenvironment (TME), affecting the tumor immune landscape and tumor response to immunotherapy (Ireland and Oliver, 2020; Teijeira et al., 2020). LncRNAs, as particular type of non-coding RNAs, have been reported to mediate NETs-induced cancer cell metastasis in the TME (Wang et al., 2022). Therefore, the exploration of the relevance of the model based on NETs-related lncRNAs and the immune microenvironment of BC patients is essential.

In this current study, we comprehensively identified NETs-related lncRNAs by constructing a co-expression network based on the correlation analysis between lncRNAs and NETs-related genes. To avoid overfitting and strengthen the clinical practicability, univariate, LASSO, and multivariable logistic regression algorithms were applied to screen out 10 NETs-related lncRNAs for the construction of prognostic risk model. Furthermore, we stratified patients into high-risk and low-risk groups based on this prognostic risk model. Survival analysis showed poorer prognosis in high-risk group. The risk score was an independent risk parameter in a cox regression analysis combining with clinical characteristics (age, stage, and subtype). Functional enrichment analysis of differentially expressed genes indicated that there were significantly different in immune-related functions and pathways between the two groups. Moreover, the tumor immune cells, immune function, immune checkpoint genes, and drug sensitivity in BC based on the prognostic risk model were further analyzed, which all demonstrated the potential predictive utility of the model in immunotherapy of BC patients. Finally, as demonstrated by our validation experiment, we were able to confirm the consistency expression of NETs-related lncRNAs by qRT-PCR in the BC tumor tissue and paired normal tissue.

After witnessing the success of molecular targeted therapies in the clinical application of several solid tumors, there is a growing enthusiasm in studying the impact of lncRNAs on tumors (Yang et al., 2022; Yardim-Akaydin et al., 2022). The lncRNA SFTA1P acts as an oncogene and promotes the growth and invasion of lung (Du et al., 2020; Zhu et al., 2021) and liver cancers (Huang et al., 2020) in various signaling pathways, such as mTOR signaling pathway, AKT signaling pathway. Interestingly, in the GSEA analysis, we also found that the mTOR signaling pathway was enriched in high-risk group. Several studies have reported the function of ACTA2-AS1, enhancing the malignant phenotype of cervical cancer (Luo et al., 2020), while exhibiting anti-tumor effects in liver cancer (Zhou and Lv, 2019), and lung adenocarcinoma (Ying et al., 2020). Additionally, ACTA2-AS1 has been implicated in platinum resistance in ovarian cancer and lung cancer (Lin et al., 2022; Liu et al., 2022). LINC01235 promotes gastric cancer migration and invasion *via* epithelial-mesenchymal transition (EMT) pathway (Zhang et al., 2021). Of note, NETs can activate the EMT program to drive the pro-metastatic phenotype of human breast cancer cells (Martins-Cardoso et al., 2020). LINC01010 is downregulated in HBV-transgenic hepatocellular carcinoma cell line and is a potential tumor suppressor that inhibits the development of HBV-associated hepatocellular carcinoma (Gan et al., 2021). Tang et al. (2021) reported that MIR200CHG can directly bind to the transcription factor YB-1 and inhibit its ubiquitination and degradation to promote proliferation, invasion, and drug resistance in breast cancer. However, in our current study, MIR200CHG acts as a protective factor in the development of BC. Therefore, further studies are needed to explore its role. For the five remaining NETs-related lncRNAs (AC004816.2, AC000067.1, AL133467.1, AC092919.1, and AL591468.1), there have been no studies exploring their potential roles in the development of cancer at present. Thus, further research in the future is needed to understand the deeper mechanisms.

NETs-related lncRNAs play a multifaceted role in the tumor microenvironment and can alter the interaction and crosstalk between tumor cells and the tumor microenvironment, leading to immunosuppression and therapy resistance, thereby allowing tumor cells to evade immune surveillance (Papayannopoulos, 2018; Yang et al., 2022). Here, the GO and KEGG analyses indicated that the differentially expressed genes between the high- and low-risk groups were mainly enriched in immune-related pathways. This further confirms the role of NETs-related lncRNAs in the tumor microenvironment by mainly influencing the immune function of the body.

Genetic mutations are the basis for tumor development, and specific mutations predict the response to therapy and prognosis (Chan et al., 2019). In our current study, PIK3CA mutations occurred most commonly between the two groups and appeared more frequently in the low-risk group than in the high-risk group. Several studies have been conducted on PIK3CA mutations, but results on the prognostic significance of PIK3CA mutations appear to be conflicting (Kalinsky et al., 2009; Mosele et al., 2020). Here, PIK3CA mutation is a positive effect on BC patient survival. Further studies on the effect of PI3K mutations on patient survival need to be investigated. TMB is emerging as a potent biomarker for predicting the efficacy of immunotherapy in cancer patients (Chan et al., 2019; Sha et al., 2020). In this study, the TMB of BC patients was positively correlated with the risk score. High-risk patients had higher levels of immune checkpoint genes expression and better immunotherapy outcomes, which indicated that the prediction result of TMB and NETs-related lncRNAs is consistent. Notably, the risk score appeared to have a greater impact on BC patient survival compared to TMB. Therefore, the risk score of BC patients can be used as a complement to TMB to better predict patient immunotherapy outcomes.

The tumor microenvironment (TME), composed of tumor cells and stromal cells, is associated with tumorigenesis, pathogenesis, and tumor progression, supporting the cancer cells replicative proliferation and affecting the tumors malignant phenotype (Chen and Song, 2022; Franzén et al., 2022). The presence of many immune cells in TME, charactered by a “double-edged sword”, behave as the anti-tumor and pro-tumor cells, protecting us against tumor cells or modulating tumor cells migration, invasion, metastasis, and anticancer drug sensitivity (Li et al., 2020; Mehraj et al., 2021; Peña-Romero and Orenes-Piñero, 2022). In this current study, the differences in immune infiltration, immune function, and immune checkpoint genes expression between high-risk and low-risk BC patients were elaborated, and we found statistically significant differences in the immune status of the body. Activated dendritic cells, activated CD8 T cells, natural killer cells and mast cells have been reported to be antitumor immune cells (Peña-Romero and Orenes-Piñero, 2022). Here, we found higher levels of tumor antagonistic immune cells in the low-risk group compared to the high-risk group. Also, immune function analysis showed that patients with low-risk scores exhibited more immune activity, which probably explains why low-risk patients have a better prognosis. Historically, breast cancer was not a highly immunogenic tumor due to the low mutation rate and few neoantigens, and selecting the suitable patients for immunotherapy is difficult (Franzén et al., 2022). In the present study, patients with high-risk scores had higher expression of immune checkpoint genes and better immunotherapy outcomes, suggesting that our signature might be used to assess the suitable population for immunotherapy.

Finally, based on the “pRophetic” algorithm and the TEDER program, we assessed the susceptibility of high- and low-risk populations to commonly used chemotherapeutic agents, molecularly targeted therapies, and immunotherapeutic responses. Our results confirm the potential predictive value of NETs-related lncRNAs for chemotherapy susceptibility, targeted therapy and immunotherapy efficacy. BC chemotherapy agents, such as cisplatin, gemcitabine, paclitaxel and vinorelbine, are more sensitive in low-risk groups. Gefitinib, an epidermal growth factor receptor (EGFR) inhibitor, is effective in cancers that have activated mutations in EGFR. However, BC routinely exhibit intrinsic resistance to anti-EGFR therapeutics (You et al., 2021). Here, we identified the sensitivity of low-risk populations to gefitinib, suggesting that anti-EGFR therapy is effective for specific populations. Lapatinib, a reversible inhibitor of intracellular tyrosine kinase activity of HER2 and EGFR1, is used in combination with capecitabine in advanced or metastatic BC (Tesch and Gelmon, 2020). In our study, the IC_50_ value of lapatinib was lower in the high-risk group, indicating that lapatinib is more appropriate for these high-risk populations. As immune checkpoint inhibitors improve overall survival in various cancers, the accompanying immunotherapy-mediated side-effects, such as colitis, hepatitis, and rheumatic diseases, have drawn attention (Kim et al., 2022; Okiyama and Tanaka, 2022). Selective the appropriate immunosuppressive agents for BC treatment are necessary due to the differences in the immune system of individuals and their sensitivity to drugs. Here, we explored the drug sensitivity of two immunosuppressive agents: methotrexate and rapamycin. Methotrexate is commonly used in the treatment of autoimmune diseases, such as rheumatic immune-related adverse events (Kostine et al., 2018). Some studies have reported that the combination of rapamycin and other chemotherapeutic agents can improve the efficacy (Niu et al., 2011; Sun et al., 2021). In addition, the combination with rapamycin and anti-PD-1 synergistically inhibits tumor growth and mitigates immune-related colitis in a mouse melanoma model (Bai et al., 2021). Thus, methotrexate and rapamycin are promising for the treatment of adverse events caused by immunotherapy. In our exploration, the IC_50_ values of methotrexate and rapamycin were higher in high-risk group. Understandably, as the efficacy of immunotherapy increases, so do the side effects that come with it. Thus, our NETs-related lncRNAs signature may help to identify patients who would benefit from immunosuppressive therapy, but the underlying mechanism of action still needs to be clarified.

Certainly, there are several potential limitations to the current study. The in-depth molecular mechanisms used to construct NETs-related lncRNAs prognostic model need to be further validated in experimental studies. Moreover, the study data were based only on the TCGA public databases, which may represent a selection bias. Therefore, further multicenter, large-scale studies are now required to better determine its clinical utility and predictive validity.

## Conclusion

To summarize, a novel prognostic model based on 10 NETs-related lncRNAs was successfully constructed, which demonstrated good predictive capacity and effectiveness for BC. Furthermore, our NETs-related lncRNAs signature was significantly correlated with TMB, tumor immune microenvironment, and anti-cancer agents, indicating that these molecular changes might explain individual differences in the treatment effectiveness. These findings may provide provides a new analytical perspective on BC treatment decisions and enhance biological understanding in BC.

## Data Availability

The datasets presented in this study can be found in online repositories. The names of the repository/repositories and accession number(s) can be found below: https://portal.gdc.cancer.gov/repository, TCGA Breast Cancer (BRCA).
